# The use of viscoelastic haemostatic assays in goal-directing treatment with allogeneic blood products – A systematic review and meta-analysis

**DOI:** 10.1186/s13049-017-0378-9

**Published:** 2017-04-13

**Authors:** Mathilde Fahrendorff, Roberto S. Oliveri, Pär I. Johansson

**Affiliations:** 1 Section for Transfusion Medicine, Capital Region Blood Bank, Copenhagen University Hospital, Rigshospitalet, University of Copenhagen, Blegdamsvej 9, DK–2100 Copenhagen, Denmark; 2 Department of Surgery, Centre for Translational Injury Research, UT Health, University of Texas Health Science Center at Houston, Houston, TX USA; 3 Center for Systems Biology. The School of Engineering and Natural Sciences, University of Iceland, Reykjavik, Iceland

**Keywords:** Bleeding, Mortality, ROTEM, TEG, Thrombelastography, Thrombelastometry

## Abstract

**Background:**

Management of the critically bleeding patient can be encountered in many medical and surgical settings. Common for these patients is a high risk of dying from exsanguination secondary to developing coagulopathy. The purpose of this meta-analysis was to systematically review and assess randomised controlled trials (RCTs) performed on patients in acute need for blood transfusions due to bleeding to evaluate the effect of viscoelastic haemostatic assay (VHA) guidance on bleeding, transfusion requirements and mortality.

**Methods:**

PubMed and EMBASE were searched for RCTs that 1) randomised patients into receiving transfusions based on either a VHA-guided (thromboelastography [TEG] or rotational thromboelastometry [ROTEM]) algorithm (intervention group) or at the clinician’s discretion and/or based on conventional coagulation tests (control group) and 2) adequately reported on the outcomes bleeding and/or transfusions and/or mortality. Data on bleeding, transfusions and mortality were extracted from each trial and included in a meta-analysis.

**Results:**

Fifteen RCTs (*n* = 1238 patients) were included. Nine trials referred to cardiothoracic patients, one to liver transplantation, one to surgical excision of burn wounds and one to trauma. One trial was conducted with cirrhotic patients, one with patients undergoing scoliosis surgery while one trial randomised treatment in post-partum females presenting with bleeding. The amount of transfused red blood cells (RBCs), fresh frozen plasma (FFP) and bleeding volume was found to be significantly reduced in the VHA-guided groups, whereas no significant difference was found for platelet transfusion requirements or mortality.

## Background

Haemorrhage remains a major cause of potentially preventable deaths worldwide. Trauma and massive transfusion is associated with coagulopathy secondary to tissue injury, hypoperfusion, dilution and consumption of clotting factors and platelets [1–9]. Patients undergoing cardiac surgery accompanied by cardiopulmonary bypass (CPB) stand a high risk of dying due to microvascular bleeding and 11% have excessive bleeding after CPB – in most cases found to be nonsurgical [10, 11]. The non-surgical bleeding risk in these patients originates in coagulopathy arisen from distortion of the haemostatic system [12, 13]. Concepts of damage control surgery in trauma have evolved, prioritizing early control of the cause of bleeding by non-definitive means, while haemostatic resuscitation seeks early control of coagulopathy [14, 15]. Haemostatic resuscitation provides transfusions with fresh frozen plasma (FFP) and platelets in addition to red blood cells (RBCs) in an immediate and sustained manner as part of the transfusion protocol for critically bleeding patients. Transfusion of RBCs, FFP and platelets in a similar proportion as in whole blood prevents both hypovolemia and coagulopathy [16, 17]. Although an early and effective reversal of coagulopathy is documented [16, 18], the most effective means of preventing coagulopathy of massive transfusion remains debated. Results from recent before-and-after studies in massively bleeding patients and one randomised clinical trial (RCT) indicate that trauma exsanguination protocols involving the early administration of plasma and platelets are associated with improved survival [19–22]. Furthermore, viscoelastic haemostatic assays (VHAs), such as thrombelastography (TEG)/rotational thromboelastometry (ROTEM), appear advantageous for identifying coagulopathy in patients with severe haemorrhage, as opposed to conventional coagulation tests (CCTs) [23–25]. Current views recommend that patients with uncontrolled bleeding, regardless of its cause, should be treated with goal-directed haemostatic resuscitation involving the early administration of plasma and platelets and the use of VHAs should be considered. The aim of goal-directed therapy should be to maintain a normal haemostatic competence until surgical haemostasis is achieved, as this appears to be associated with reduced mortality [4, 6, 12, 20].

The aim of the present study was to perform a systematic review and meta-analysis of all published RCTs comparing the effect of VHAs versus CCTs on blood loss, transfusion requirements and mortality.

## Materials and methods

An electronic search was conducted by one of the authors (MF) in the PubMed and EMBASE database using the following search strategy: (Thrombelastography OR Thromb?elastograph* OR thromboelastograph OR ROTEM OR TEG OR ROTEG OR Thromboelastometry OR (algorithm AND bleeding)) AND ((randomized controlled trial OR controlled clinical trial) OR (randomized OR placebo OR trial)), to identify all RCTs done on bleeding patients using treatment algorithms based on results from either TEG or ROTEM. The search identified 1245 references in PubMed and 1835 references in EMBASE. 222 duplicate findings were discarded, leaving a total of 2858 references for further assessment. References were assessed by one of the authors (MF) and discussed and consensus reached with all authors in doubt cases. Only published RCTs were eligible for this analysis. Inclusion criteria were 1) trial designs in which patients were randomly allocated to receive transfusions based on either a VHA-guided (TEG or ROTEM) algorithm (intervention group) or at the clinician’s discretion and/or based on laboratory coagulation tests (control group) and 2) references had to adequately report the outcomes bleeding and/or transfusions and/or mortality. Studies written in other languages than English were also eligible for inclusion. Trials were excluded immediately based on title or abstract, if they did not meet the inclusion criteria. Moreover, trials that were not performed on humans and paediatric studies were also excluded. The remaining studies were evaluated and assessed for relevance by all authors. Reference lists of the included studies were searched for subsequent relevant studies not identified by search engines. Corresponding authors were contacted to retrieve inadequately reported or missing data. Primary outcomes for data extraction were all-cause mortality, total amount of bleeding expressed either as bleeding at 12 h, 24 h or perioperative amount of bleeding and amount of total RBC transfusions, FFP transfusions and platelet transfusions. When amount of blood transfusions was given in mL, calculations of the corresponding number of units were done using the conversion factors illustrated in table 1. The volume per unit was an estimate of the standard volume of the given allogeneic blood product over the last years in the Capital Region Blood Bank, Rigshospitalet, Copenhagen. The latest follow up data on mortality were used in the analysis of all-cause mortality.

**Table 1:** Conversion factors from mL to units

1U RBC	250 mL/U
1U FFP	270 mL/U
1U platelet concentrate	340 mL/U

## Statistics

Statistical meta-analyses were conducted using Review Manager (RevMan) Version 5.3 (The Nordic Cochrane Centre, The Cochrane Collaboration, 2014). Pooled estimates and their 95% confidence intervals (CI) were calculated using the inverse variance method. The random-effects model was used in anticipation of significant heterogeneity [26]. Statistical heterogeneity was explored using the inconsistency (I^2^) measure [27]. For all calculations, two-tailed P values of less than 0.05 were considered statistically significant.

## Results

### Study characteristics

**Fig. 1:**
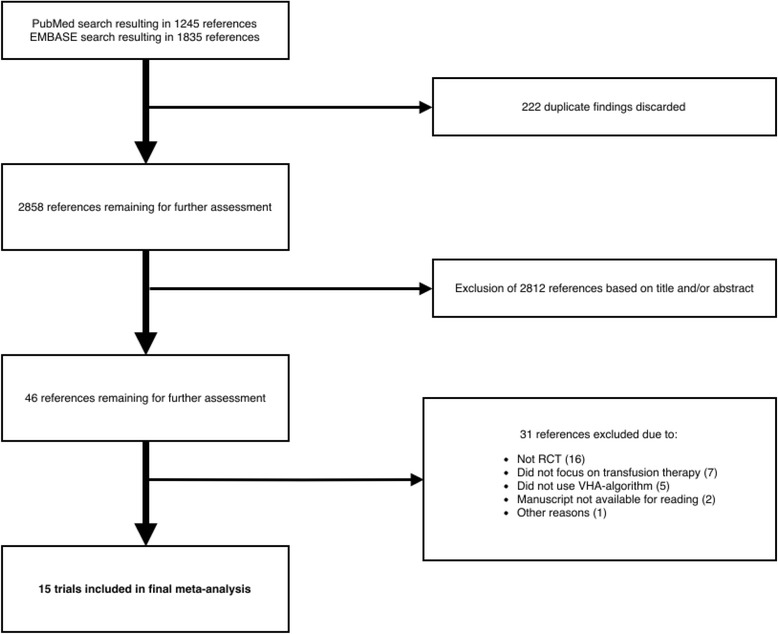
Process of inclusion of trials into meta-analysis

We identified a total of 2858 references. All references were screened by their title and abstract and 2812 references were found not to be relevant for this meta-analysis and were therefore excluded immediately, leaving 46 references for further scrutiny (fig. 1). Another 31 references were excluded due to the reasons explained in table 2. This left 15 RCTs with a total of *n* = 1238 patients to be included in this analysis. Of these trials, 9 referred to cardiothoracic patients [28–36] and one each to liver transplantation [37], surgical excision of burn wounds [38], trauma [22], cirrhotic patients [39], scoliosis surgery [40] and post-partum haemorrhage [41]. In twelve studies the intervention group was guided by TEG [22, 28, 29, 31–35, 37, 39–41] and in the remaining three by ROTEM [30, 36, 38]. Seven trials applied both results from CCTs and the discretion of the attending physician to guide the transfusions of the control group [28, 31, 32, 35, 38, 40, 41], while the control groups of eight trials were guided only by CCTs [22, 29, 30, 33, 34, 36, 37, 39] with the first transfused blood products being guided solely at the clinician’s discretion before blood analyses were available in two trials [22, 30]. Eleven trials reported on bleeding [28–35, 37, 40, 41], nine reported on mortality [22, 28, 30, 33, 34, 37, 39–41] and all studies reported on transfusion requirements. The transfusion triggers for RBCs, FFP and platelet concentrates for each study are demonstrated in table 3 and the individual study characteristics are presented in table 4.

**Fig. 2:**
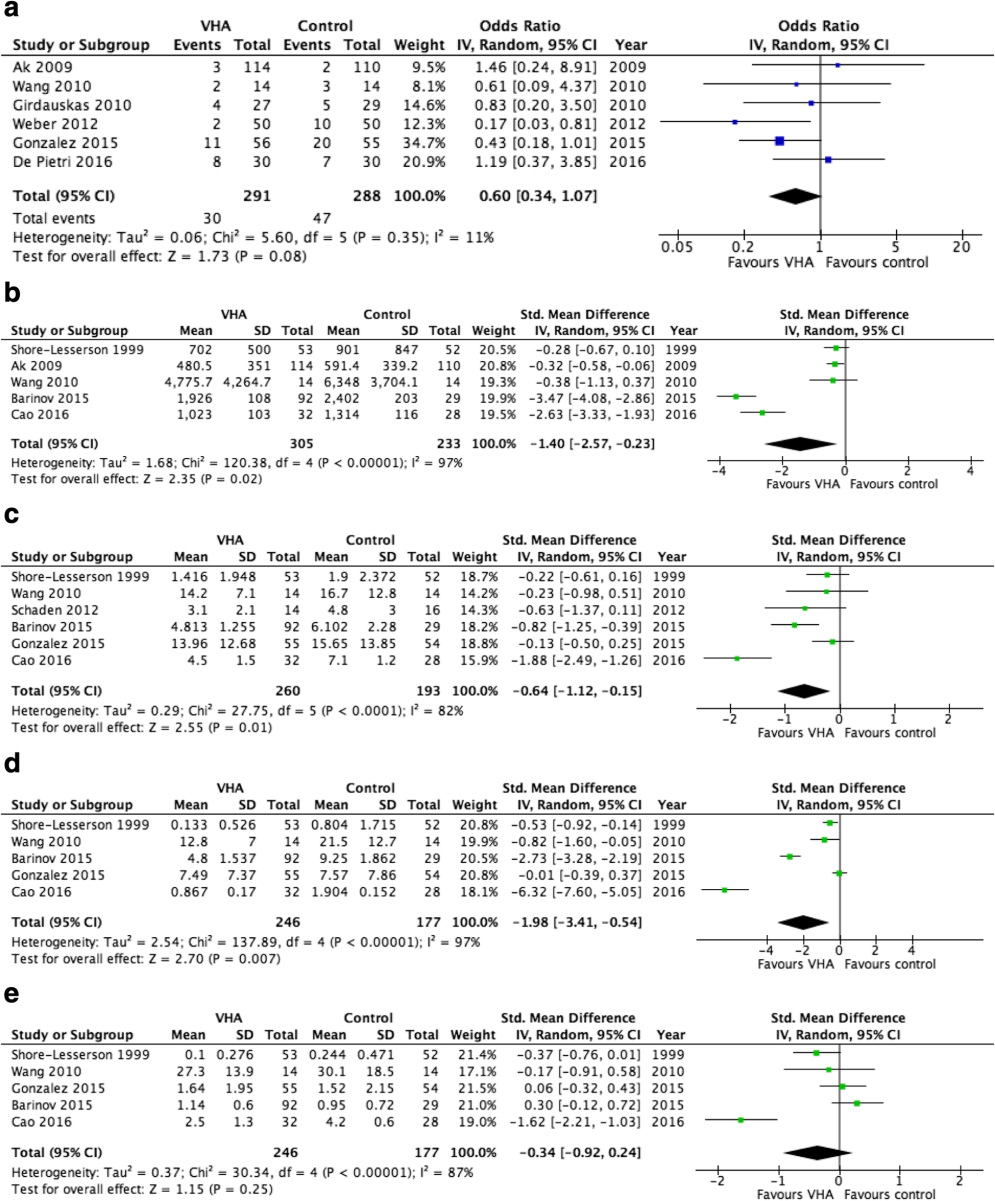
Forest plots **a** All-cause mortality **b** Perioperative, 24 h and 12 h bleeding **c** Total transfusion need – RBC **d** Total transfusion need – FFP **e** Total transfusion need – Platelets

**Table 2:** Author and year, type of patients examined and reason for exclusion in excluded scrutinized references

Reference (Author and year)	Condition	Reason for exclusion
Agarwal 2015 [46]	Cardiac surgery	Focus on platelet function testing
Branco 2014 [47]	Trauma	Observational trial
Brilej 2016 [48]	Trauma	Observational trial
Capraro 2001 [49]	Cardiac surgery	No use of VHA
Despotis 1994 (a) [50]	Cardiac surgery	No use of VHA
Despotis 1994 (b) [51]	Cardiac surgery	No use of VHA
Dietrich 2008 [52]	Cardiac surgery	Focus on TXA-therapy
Einersen 2016 [53]	Trauma	Observational trial
Hajek 2010 [54]	Cardiac surgery	Intervention group is managed both with CCT and VHA-analyses
Hanke 2012 [55]	Aortic surgery	Not randomised – matched control group
Harding 1997 [56]	Liver transplantation	Observational trial
Helm 1998 [57]	Cardiac surgery	Not randomised – matched control group
Hoenicka 2015 [58]	Cardiac surgery	Focus on heparin management
Hopkins 1983 [59]	Acute hypotension	General treatment algorithm
Israelian 2009 [60]	Neuro surgery	Possibly relevant. Manuscript not available for reading. Contact information of corresponding author not available.
Karkouti 2016 [61]	Cardiac surgery	Stepped-Wedge Clustered RCT
Levin 2014 [62]	Cardiac surgery	Focus on protamine-administration
Lier 2009 [63]	Trauma	Review
Mallaiah 2015 [64]	Obstetric haemorrhage	Before-after trial
Manikappa 2011 [65]	Cardiac surgery	Whole blood transfusions
Messenger 2011 [66]	Trauma	Prospective cohort study
Mishra 2015 [67]	Cardiac surgery	Focus on platelet function testing
Naik 2015 [68]	Major spinal surgery	Non-randomised
Petricevic 2013 [69]	Cardiac surgery	Observational trial
Rahe-Meyer 2009 [70]	Aortic surgery	Non-randomised
Roullet 2015 [71]	Orthotopic liver transplantation	Non-randomised
Smart 2015 [72]	Orthotopic liver transplantation	Retrospective non-randomised trial
Stancheva 2011 [73]	Orthotopic liver transplantation	Observational trial
Tarabarin 2013 [74]	Bile duct surgery	Possibly relevant. Manuscript not available for reading. Contact information of corresponding author not available.
Weitzel 2012 [75]	Cardiac surgery	Focus on platelet function
Xu 2014 [76]	Cardiac surgery	Focus on platelet function testing

*VHA* viscoelastic haemostatic assay, *TXA* tranexamic acid

**Table 3:** Transfusion algorithm trigger values. Table explaining individual transfusion trigger values in the respective trials included in the meta-analyses

Reference (Author and year)	RBC		FFP		Platelets		Other	
	Control group	Intervention group	Control group	Intervention group	Control group	Intervention group	Control group	Intervention group
Shore-Lesserson 1999 [33]	Hct < 25% (during CPB <21%)	Hct < 25% (during CPB <21%)	PT >150% of control (2U FFP)	hTEG R > 20 mm (2U FFP)	PC < 100 · 10^3^/μL (6U PC)	PC < 100 · 10^3^/μL AND TEG MA < 45 mm (6U PC)	Fibrinogen <100 mg/dL 10U of cryo EACA 10 g if failure	Fibrinogen <100 mg/dL 10U of cryo LY30 > 7.5% EACA 10 g
Nuttall 2001 [31]	N/A	N/A	Clinician’s discretion with or without CCT	POC PT > 16.6 s and/or POC APTT > 57 s	Clinician’s discretion with or without CCT	PC < 102 · 10^3^/mm^3^ and/or TEG MA <48 mm (PC or DDAVP)	Clinician’s discretion with or without CCT	Fibrinogen <144 mg/dL – cryo
Royston 2001 [32]	N/A	N/A	Clinician’s discretion with or without CCT	R > 14 mm < 21 mm – 1 FFP R > 21 mm < 28 mm – 2 FFP R > 28 mm – 4 FFP	Clinician’s discretion with or without CCT	MA < 48 mm – 1 platelet pool MA < 40 mm 2 platelet pools	Clinician’s discretion with or without CCT	LY30 > 7.5% - Aprotinin
Avidan 2004 [29]	Hb < 8 g/dL	Hb < 8 g/dL	If still bleeding >100 mL/h after aprotinin + desmopressin AND INR or APTT ratio > 150% control – 4U FFP	Excessive bleeding + R > 10 min – 4U FFP	Persisting excessive bleeding OR PC < 50x10^9^/L – 1 platelet pool	PFA-100® ADP channel > 120 s, epinephrine channel > 170 s treated with DDAVP 0.4 μg/kg – if bleeding persisted 1 platelet pool	Bleeding >100 mL/h within 24 h after surgery – Aprotinin (2 Mu) + desmopressin (0.4 μg/kg)	LY30 > 7.5% + bleeding >100 mL/h – aprotinin 2Mu PFA-100® ADP channel > 120 s, epinephrine channel > 170 s – DDAVP 0.4 μg/kg
Ak 2009 [28]	Htc < 25% (during CPB <18%)	Htc < 25% (during CPB <18%)	PT > 14 s or APTT > 150% normal	R > 14 mm <21 mm – 1 FFP R ≥ 21 mm <28 mm – 2 FFP R ≥ 28 mm – 4 FFP	PC < 100 · 10^3^/μL	40 ≤ MA < 48 mm – 1U platelets MA < 40 mm 2U platelets	Absence of visible clots + presence of generalized oozing-type bleeding in surgical field – TXA	LY30 > 7.5% - TXA
Westbrook 2009 [35]	Clinician’s discretion with CCT	Hb > 70 g/L	Clinician’s discretion with CCT	11 min < R(H) ≤ 14 min – 1U FFP 14 min < R(H) ≤ 20 min – 2U FFP 200 min < R(H) – 4U FFP	Clinician’s discretion with CCT	MA(H) ≤ 41 mm – 5U platelets	TXA according to clinician’s discretion with CCT	LY30 > 15% - TXA
Girdauskas 2010 [30]	Htc < 25% (Hb 8.5 g/dL) (during CPB Htc < 20% (Hb 6.8 g/dL)) or physiologic transfusion triggers	Htc < 25% (Hb 8.5 g/dL) (during CPB Htc < 20% (Hb 6.8 g/dL)) or physiologic transfusion triggers	PT > 60s or INR >1.5 – FFP 15 mL/kg body mass	HEPTEM CT > 260 s – FFP 15 mL/kg body mass	PC < 100 · 10^3^/μL – 1 platelet concentrate	(A) HEPTEM MCF 35-45 mm – 1 platelet concentrate (B) FIBTEM MCF >8 mm and HEPTEM MCF <35 mm – 1 platelet concentrate	Fibrinogen <1.2 mg/dL – 2 g fibrinogen α_2_-Antiplasmin <80% - 3 g TXA	FIBTEM <8 mm – 2 g fibrinogen APTEM MCF/HEPTEM MCF >1.5 – 3 g TXA APTEM CT > 120 s – 3000 IU PPSB
Wang 2010 [37]	Hb <8 g/dL	Hb <8 g/dL	PT and aPTT > 150% control	R > 10 min	PC < 50x10^9^/L	MA < 55 mm – 6-8U pooled platelets	Fibrinogen <1 g/dL – cryo	α-angle < 45° - cryo
Paniagua 2011 [36]	N/A	N/A	N/A	N/A	N/A	N/A	N/A	N/A
Schaden 2012 [38]	Hb <8 g/dL	Hb <8 g/dL	Clinician’s discretion with or without CCT	EXTEM CT > 100 s – 4U FFP	Clinician’s discretion with or without CCT	EXTEM A10 < 45 mm and FIBTEM >12 mm – 1U platelets concentrate	TXA and fibrinogen according to clinician’s discretion with or without CCT	EXTEM A10 < 45 mm and FIBTEM A10 < 12 mm – 2 g fibrinogen Spindle shaped trace APTEM A10> > EXTEM A10 – 10 mg/kg TXA EXTEM LY30 > 10% - 10 mg/kg TXA
Weber 2012 [34]	Hb <8 g/dL (during CPB Hb <6 g/dL) or physiologic transfusion triggers	Hb <8 g/dL (during CPB Hb <6 g/dL) or physiologic transfusion triggers	Transfused ≥4U PRBCs without new lab results – 15 mL/kg FFP INR > 1.4 or aPTT > 50s – 20-30 IU/kg prothrombin complex concentrate or 15 mL/kg FFP	EXTEM CT > 80s or HEPTEM >240 s – 20-30 IU/kg prothrombin complex concentrate or 15 mL/kg FFP	PC < 80000/μL	EXTEM A10 ≤ 40 mm and FIBTEM A10 > 10 mm or TRAP < 50 AU and/or ASPI <30 AU and/or ADP < 30 AU (second choice)	Fibrinogen pre-value < 200 mg/dL or currently <150 mg/dL – 25-50 mg/kg fibrinogen Suspected platelet dysfunction – 0.3 μg/kg desmopressin	FIBTEM MCF = 0 mm – 25 mg/kg fibrinogen before protamine EXTEM A10 ≤ 40 mm and FIBTEM A10 ≤ 10 mm – 25-50 mg/kg fibrinogen TRAP < 50 AU and/or ASPI <30 AU and/or ADP < 30 AU – 0.3 μg/kg desmopressin (first choice)
Barinov 2015 [41]	N/A	N/A	Clinician’s discretion with CCT	N/A	Clinician’s discretion with CCT	N/A	Clinician’s discretion with CCT	N/A
Gonzalez 2015 [22]	First units of RBC administered according to clinician’s discretion only Hb < 10 g/dL	First units of RBC administered according to clinician’s discretion only Hb < 10 g/dL	First units of FFP administered according to clinician’s discretion only INR ≥ 1.5 – 2U FFP	First units of FFP administered according to clinician’s discretion only ACT 111-139 s – 2U FFP ACT ≥ 140 s – 2U FFP, 10-pack cryo and 1U apheresis platelets ACT > 110 s – 2U FFP	PC < 100 · 10^3^/μL – 1U apheresis platelets	ACT ≥ 140 s – 2U FFP, 10-pack cryo and 1U apheresis platelets MA < 55 mm – 1U apheresis platelets	Fibrinogen >150 mg/dL – 10-pack cryo Suspicion on fibrinolysis with D-dimer >0.5 μg/dL – 1 g TXA	ACT ≥ 140 s – 2U FFP, 10-pack cryo and 1U apheresis platelets α-angle < 63° - 10-pack cryo LY30 ≥ 7.5% - 1 g TXA (after 61% of enrolment LY30 ≥ 3% - 1 g TXA)
De Pietri 2015 [39]	Hb <8 g/dL	Hb <8 g/dL	INR > 1.8 – 10 mL/kg ideal body weight	R > 40 min – 10 mL/kg ideal body weight	PC < 50 · 10^9^/L – 1U PLT	MA < 30 mm – 1U apheresis platelets		
Cao 2016 [40]	Hb < 70 g/L, Htc < 25% - 2U RBC	Hb < 70 g/L, Htc < 25% - 2U RBC	Clinican’s discretion	R > 8 min – FFP 15 mL/kg	PC < 50 · 10^9^/L – 1U PLT	MA < 70 mm – 1U platelets	Fibrinogen < 0.0012 mg/L – fibrinogen 2 g	α-angle < 72° - fibrinogen 2 g

Control group = groups managed without the use of either TEG or ROTEM. Intervention group = groups managed with the use of TEG or ROTEM. *Htc* haematocrit, *Hb* haemoglobin, *PC* platelet count, *U* units, *PT* prothrombin time, *N/A* not applicable, *CCT* conventional coagulation test, *RBC* red blood cell, *FFP* fresh frozen plasma, *PLT* platelets, *INR* international normalized ratio, *ACT* activated clotting time, *MA* maximal amplitude, *TXA* tranexamic acid, *R* reaction time, *aPTT* activated partial thromboplastin time, *CPB* cardiopulmonary bypass, *hTEG* heparinase-TEG, *POC* point of care

**Table 4:** Study characteristics Author and year, number of patients allocated to control or intervention group and the type of patients and/or procedures performed during the study

Reference (Author and year)	Control/intervention (n)	Type of patients/procedures
Shore-Lesserson 1999 [33]	52/53	Cardiac surgery Moderate to high risk of microvascular bleeding (single/multiple valve replacement, combined CAB + valvular procedure, cardiac reoperation, thoracic aortic replacement). CPB performed with moderate hypothermia.
Nuttall 2001 [31]	51/41	Cardiac surgery All types of elective cardiac surgery developing abnormal bleeding after CPB.
Royston 2001 [32]	30/30	Cardiac surgery 10% in each group had heart transplantation, 50% in each group had revascularization (multiple grafts with an estimated CPB-time >100 min), 40% in each group Ross procedure, multiple valve or valve and revascularization surgery.
Avidan 2004 [29]	51/51	Cardiac surgery Routine elective first time coronary artery surgery with CPB. Cooled to 32 °C.
Ak 2009 [28]	110/114	Cardiac surgery Elective first time coronary artery bypass graft (CABG) with CPB.
Westbrook 2009 [35]	37/32	Cardiac surgery Presenting for cardiac surgery except lung transplantations.
Girdauskas 2010 [30]	29/27	Aortic surgery Patients undergoing aortic surgery with hypothermic circulatory arrest. 25 patients with acute type A dissection.
Wang 2010 [37]	14/14	Orthotopic liver transplantation
Paniagua 2011 [36]	9/13	Cardiac surgery Patients scheduled for cardiac surgery with extracorporeal circulation with major post-operative bleeding (>300 mL).
Schaden 2012 [38]	16/14	Surgical excision of burn wounds Surgical intervention performed on 3rd day after trauma.
Weber 2012 [34]	50/50	Cardiac surgery Patients scheduled for elective, complex cardiothoracic surgery (combined coronary artery bypass, graft and valve surgery, double/triple valve procedures, aortic surgery or redo surgery) with CPB.
Barinov 2015 [41]	29/90	Postpartum obstetric haemorrhage
Gonzalez 2015 [22]	55/56	Trauma patients Meeting criteria for massive transfusion protocol (MTP) activation on arrival to ED: systolic blood pressure <70 mmHg or SBP 70 – 90 mmHg with heart rate 108 beats/min, in addition to any of the following injury patterns: penetrating torso wound, unstable pelvic fracture, or abdominal ultrasound suspicious of bleeding in more than one region.
De Pietri 2015 [39]	30/30	Hepatic surgery Patients with cirrhosis + significant coagulopathy (defined as INR >1,8 and/or platelet count <50 × 109/L) undergoing invasive procedure.
Cao 2016 [40]	28/32	Scoliosis surgery Patients with an expected surgical bleeding > 1000 ml and the American Society of Anesthesiologists rating I-II in addition to a body mass index (BMI) 18 to 24 kg/m^2^

*CAB* coronary arterial bypass, *CABG* coronary artery bypass graft, *CPB* cardio pulmonary bypass, *MTP* massive transfusion protocol, *ED* emergency department, *SBP* systolic blood pressure, *INR* international normalised ratio

### Meta-analyses

#### All-cause mortality

Six trials were included in the meta-analysis of all-cause mortality with a total of 579 patients of whom 291 patients were allocated to the intervention. Three trials concerned patients undergoing cardiothoracic surgery [28, 30, 34] one trial concerned orthotopic liver transplantation [37], one studied cirrhotic liver patients [39] and one studied trauma patients [22]. The meta-analysis demonstrated no difference in survival between the groups with an OR of 0.60 (95% CI 0.34 to 1.07; *p* = 0.08) (figure 2a).

#### Bleeding volume

Eleven RCTs reported on bleeding while only five of these studies expressed perioperative, 24 or 12-h bleeding as mean ± SD and were therefore eligible for meta-analysis [28, 37, 40–42]. Comparison of the bleeding volume in 538 patients (305 in the intervention groups) resulted in significantly reduced bleeding in the VHA treated patients (standardized mean difference −1.40 [95% CI 2.57 to −0,23]; *p* = 0.02) (figure 2b).

#### Transfusion requirements

The analysis for transfusion requirements was limited to six trials concerning RBC transfusions [22, 37, 38, 40–42] and five trials were eligible for the meta-analysis on transfusions of FFP and platelets, respectively [22, 37, 40–42]. All fifteen trials included in this analysis reported on transfusions, while only the above mentioned described the mean transfused amount per patient ± SD as required for meta-analysis. Isolating RBC-transfusion requirements, 260 out of 453 patients were in the intervention group. Random effects analysis resulted in a standardized mean difference of −0.64 (95% CI −1.12 to −0.15; *p* = 0.01), being statistically significant (figure 2c). Differences in FFP-transfusions were calculated in 423 patients (246 in intervention group) and resulted in a standardized mean difference of −1.98 (95% CI −3.41 to −0.54; *p* = 0.007), showing a significant reduction in transfused FFP in the intervention group (figure 2d). Numbers for transfused units of platelets were available from the same 423 patients as with FFP-transfusion requirements, however meta-analysis did not reach statistical significance (standardized mean difference −0.34 [95% CI −0.92 to 0.24; *p* = 0.25]) (figure 2e).

## Discussion

We found the total bleeding volume and the amount of transfused RBCs and FFP to be significantly reduced in the VHA-guided intervention groups compared to CCT-guided control groups. Considering that most trials used the same transfusion trigger for RBCs in both groups, the difference in RBC requirements may be explained by a better haemostatic competence in TEG/ROTEM-guided groups accomplished through timely administration of plasma and platelets, further supported by the reduction of bleeding in the VHA-guided group of patients. In our meta-analysis no statistically significant difference was found between groups regarding all cause-mortality and required amounts of platelets. The sizes of the respective trial populations were small and a lack of cohesion in permission of platelet inhibitors, anticoagulants, antifibrinolytics and triggers used to guide resuscitation with blood products was observed. The control groups were managed either by clinical judgement combined with CCTs or by the sole use of algorithms applying only CCT-triggers for transfusion. The decision to transfuse potentially encompasses a bias to a greater number of transfusions between clinicians with a different background and clinical practice, in alignment with Avidan et al. [29] finding a reduction in transfusions administered with CTT-algorithm guided perioperative management versus transfusion guidance based only on the physician’s discretion. Although only a difference in amount of FFP and no statistical difference in the amount of platelets transfused between groups was detected, the timing of these transfusions may differ with VHA-analyses having shorter turn-around time than conventional coagulation tests [43]. This accentuates the importance of early administration of the appropriate blood products as also emphasized by Cotton et al. [20] who found reduced odds of mortality (74%) and transfusions in a group of trauma patients managed with early and aggressive resuscitation on admittance to the emergency department. Although 24-h transfusion requirements were reduced in patients treated according to the exsanguination protocol, amounts of intraoperative transfusions were found to be larger in this cohort in comparison with the conventionally treated controls, illustrating the importance of early resuscitation with blood products. Also Johansson et al. [21] found similar results in patients undergoing surgery for ruptured abdominal aortic aneurysm (rAAA) with a proactive intraoperative administration of platelets and FFP yielding an increase in survival in massively bleeding rAAA patients. They found a significant reduction in postoperative transfusions, indicating that early blood product administration plays a pivotal role in improving haemostasis in massive bleeders. Gonzalez et al. [22] have conducted the first RCT to evaluate VHA-guided transfusion therapy in trauma. They found a survival benefit in the TEG-guided group especially with regards to less haemorrhagic and early deaths. Additionally, they argued that the administration of more platelets and FFP does not necessarily increase survival chances but highlight the effect of the appropriate treatment being given at the optimal time rather than the amount of blood product administered. Moreover, in patients undergoing surgery with extracorporeal circulation, the use of TEG/ROTEM heparinase analyses, where coagulopathy can be identified despite patient being heparinized, may provide an even earlier assessment of coagulation status and thereby enable an earlier correction of coagulopathies, exemplified by Royston et al. [32] and Girdauskas et al. [30].

Weber et al. [34] report a notably higher mortality among their patients than usually seen in cardiac surgery. Despite this, we did not find a significant difference in mortality in the VHA-guided groups compared to conventionally treated groups. However, our meta-analysis suggested clinical difference in survival in patients having treatment based on VHA-results, in congruence with a before- and after study conducted on trauma patients by Johansson et al., showing a reduction in mortality of approximately 30% in a group resuscitated using TEG results in patients requiring massive transfusions [19]. Furthermore, a Cochrane review from Wikkelsø et al. [44] found the use of TEG or ROTEM in guiding resuscitation of bleeding patients to reduce all-cause mortality and the number of patients transfused with blood products, although no difference was found with regard to excessive bleeding events and proportion of massively transfused, in agreement with our results. Also, NICE-report done by Whiting et al. [45] finds a tendency to fewer transfusions of allogeneic blood products being administered in cardiac surgery patients treated according to VHA-results when comparing to patients managed with CCT-results, while no difference was found with regard to trauma patients and post-partum bleeding. The discrepancies in study selection with the review from Whiting et al. [45] are explained in table 5.

**Table 5:** Explanation for discrepancies with RTCs included by Whiting et al. [45] (NICE-report)

Reference (author and year)	Reason for exclusion from this meta-analysis
Kultufan Turan et al. 2006	Not possible to identify in PubMed or EMBASE
Rauter et al. 2007	Not possible to identify in PubMed or EMBASE
Messenger et al. 2011	Prospective cohort study, not randomised

## Limitations

A limited number of adequately reported trials were eligible for our meta-analyses. Out of the 15 included trials in this analysis, five did not report sufficient information to be included in any of the meta-analyses performed [29, 31, 32, 35, 36]. This meta-analysis has an overweight of trials concerning cardio-thoracic patients, while other patient groups are only represented by a single RCT each, limiting comparability of results. Furthermore, the studies included present patients with bleeding originating from different aetiologies. This can potentially be problematic in that the severity of bleeding may vary.

## Conclusions

In conclusion, the performed meta-analyses demonstrated trends towards the superiority of treating haemorrhaging patients under the guidance of VHA-algorithms. There is, however, a need for larger RCTs, such as the ongoing trials “implementing Treatment Algorithms for the Correction of Trauma Induced Coagulopathy (iTACTIC)” NCT02593877.

## Abbreviations

CCT: Conventional coagulation test
CI: Confidence intervals
FFP: Fresh frozen plasma
iTACTIC: Implementing Treatment Algorithms for the Correction of Trauma Induced Coagulopathy
rAAA: Ruptured abdominal aorta aneurism
RBCs: Red blood cells
RCT: Randomised controlled trial
ROTEM: Thromboelastometry
SD: Standard deviation
TEG: Thromboelastography
VHA: Viscoelastic haemostatic assay
